# Randomized Controlled Trial Comparing Ferrous Sulfate and Iron Sucrose in Iron Deficiency Anemia in Pregnancy

**DOI:** 10.7759/cureus.34858

**Published:** 2023-02-11

**Authors:** Neha Chauhan, Poojan Dogra, Reena Sharma, Shashi Kant, Mridul Soni

**Affiliations:** 1 Obstetrics and Gynaecology, Shri Lal Bahadur Shastri Government Medical College & Hospital, Mandi, IND; 2 Obstetrics and Gynaecology, All India Institute of Medical Sciences, Bilaspur, IND; 3 Research, Shri Lal Bahadur Shastri Government Medical College & Hospital, Mandi, IND

**Keywords:** serum ferritin, hemoglobin, iron deficiency anemia (ida), nutrition of pregnant women, anemia

## Abstract

Introduction: Anemia among pregnant women is one of the major health concerns for healthcare workers. The management becomes a concern in the pregnancy where the question arises of which is better the intravenous iron sucrose or the oral ferrous sulfate tablets. To answer this, a randomized control trial comparing both the treatment options in a tertiary care government hospital was set up in the hilly terrains of India. This study discusses the effectiveness and practical aspect of using both, which seems to be the better out of both, and why.

Methods: The study was conducted as a parallel-group, open-label randomized controlled trial (RCT) in the Department of Obstetrics and Gynecology of a tertiary care government hospital in India, with approximately 4,000 delivery loads annually. Ethical clearance was obtained from the institute’s ethics committee (IEC), and the trial was registered with the Clinical Trial Registry of India (REF/2022/06/055013). Two hundred sixty-eight pregnant women between 18 and 45 years of age with moderate iron deficiency anemia (IDA) (hemoglobin (Hb) 7-9g/dl, microcytic-hypochromic, and serum ferritin <30ng/ml) were included in the study. Patients were randomly divided into two groups: group 1 with 134 patients to receive intravenous iron sucrose and group 2 with 134 patients to receive oral ferrous sulfate tablets.

Results: The intravenous iron sucrose is superior in terms of tolerability and correction of iron deficiency anemia during pregnancy.

Conclusion: It yields a quicker rise in Hb and serum ferritin with no major side effects. In the difficult terrain of Himachal Pradesh, this makes IV iron sucrose a better option for anemic pregnant women who do not have easy access to health facilities resulting in a large number of them reaching hospitals with moderate to severe anemia at a later gestation.

## Introduction

Anemia among pregnant women is a serious global health concern. Iron deficiency anemia (IDA) attributes to 50% of cases [1]. In India, as per the National Family Health Survey 5 (NFHS-5) from 2019 to 2021, the prevalence of anemia among pregnant women is 52.2% compared to 50.4% in NFHS-4 (2015-2016) [2-3]. In the state of Himachal Pradesh, this prevalence has come down to 42.2% compared to 50.4%; however, anemia remains a challenge for healthcare providers among pregnant women [2]. About half of the global maternal deaths due to anemia occur in South Asian countries, and India contributes to about 80% of it [4]. The condition gets aggravated during pregnancy due to the increased demand for the growing fetus and physiological changes during pregnancy [5].

Indian Council of Medical Research defines anemia as hemoglobin (Hb) less than 11 g/dl (gram per deciliter) during pregnancy and further classifies it into mild, moderate, severe, and very severe if Hb levels are < 11g/dl, 7-9g/dl, 4-7g/dl, and <4g/dl, respectively [6]. Iron deficiency anemia during pregnancy increases the risk of low birth weight, preterm birth, perinatal and maternal mortality and postpartum hemorrhage, and poor Apgar score [7]. In a systematic review, a dose-responsive relationship was observed between an increase in the dose of iron supplements and the reduction in low birth weight of newborns [8]. The Anemia Mukt Bharat strategy was initiated in 2018 to reduce the prevalence by 3% points annually [9]. It targets children aged six months to five years, adolescent girls, boys, women of reproductive age (15-49 years), and pregnant and lactating mothers [9]. The standard model of drug administration during pregnancy is oral iron. It is not associated with a hospital stay or any immediate life-threatening adverse drug reaction like anaphylactic reaction, thus requiring no monitoring unlike injectable iron preparations [10]. The main issue with oral iron therapy is a lack of education and compliance due to associated gastrointestinal side effects like bloating, diarrhea, heartburn, nausea, constipation, and dark stools [11].

Parenteral preparations were introduced to overcome these problems. Among parenteral iron preparations, intramuscular iron was associated with side effects like pain at the injection site, a dark discoloration of the skin, myalgia, and arthralgia and therefore is not much used nowadays. Intravenous parenteral preparations came into use for the treatment of anemia. They obviate the need for transfusion in the antenatal and postpartum periods [12]. Initially, iron dextran was used for several years but was associated with adverse drug reactions in 26% of patients receiving therapy. Around 0.1% to 0.6% of cases of severe anaphylaxis were also seen, after which it was banned from the market [13]. Currently, the most commonly used preparation is the iron sucrose complex [13]. Chemically it is a polymer of two main molecules, sucrose and iron (iii) hydroxide which are in solution together but are not bound [14]. When used for medical purposes, the iron complex is polymerized and sucrose molecules combine to form larger polysaccharides [14]. This is a safe drug and requires no test dose. The only disadvantage is the limited dose per sitting [14].

Despite the standard care of anemia with oral iron supplementation, most of these women remain anemic at term and delivery. This requires aggressive intervention at correcting IDA in this group early enough for the women to undergo safe delivery and decrease the likelihood of complications. Therefore, in the hilly terrains of Himachal Pradesh where following up on patients remains a challenge, the current study is undertaken to find a suitable drug among the oral standard care and intravenous iron sucrose to correct IDA in pregnant women without increasing the hospital visits and ensuing rapid correction of stores.

## Materials and methods

The study was conducted as a parallel-group, open-label randomized controlled trial (RCT) in the Department of Obstetrics and Gynaecology of Shri Lal Bahadur Shastri Government Medical College & Hospital, Mandi, Himachal Pradesh, located at a height of 2,740 feet or 835m above ground level with approximately 4,000 delivery loads annually. Ethical clearance was obtained from the institute’s ethics committee (IEC), and the trial was registered with the Clinical Trial Registry of India (REF/2022/06/055013). All pregnant women attending the antenatal clinic at 18-22 weeks gestation were screened with Hb, peripheral blood smear, RBC indices, serum ferritin, urine albumin, sugar and microscopy, and stool for ova and cyst to rule out causes of anemia. Women with Hb 7-9g/dl with IDA were enrolled for recruitment after they satisfied the inclusion and exclusion criteria.

The inclusion criteria were age between 18 and 45 years and singleton pregnancy. The pregnant women with a gestational age of 18-22 weeks have a moderate IDA with Hb levels of 7-9g/dl; when the peripheral smear was done, it revealed microcytic-hypochromic anemia with the serum ferritin (<30ng/ml).

The exclusion criteria were pregnant women with anemia other than IDA and those with a known history of allergy to injection iron. Multiple pregnancies were also excluded due to the previous iron drug intakes already ongoing making the study less understanding of the iron drug delivery method. Women with other diseases such as hypertension, diabetes mellitus, chronic liver disease, renal disorders, cardiovascular diseases, thyroid disorders, intestinal resection, bypass, hemosiderosis, and hemochromatosis could interfere with the data of the study. Informed written consent was taken from all the patients before recruitment in the study.

The sample size was taken assuming the superiority margin at 5% (i.e., delta (d)=0.05). The true difference in mean Hb between treatment groups was 0.09 (i.e., d=0.09) and the standard deviation of 12% (i.e., SD=0.12). For achieving 80% power (i.e., 1-β=0.8) at the 5% level of significance (i.e., α=0.05) with the equal allocation (i.e., k=1), the sample size for the treatment and control group was calculated to be 112. With a drop-out rate of 20%, the sample size taken was 134 in each group. A total of 268 patients were recruited in the study. The equation used for calculating sample size is as follows:

n = {Z (1-α) + Z (1-β)}^2^ × SD^2^

Two hundred sixty-eight women who fulfilled the inclusion and exclusion criteria were randomized by a computer-generated block randomization table into two groups in a 1:1 ratio. They were administered either ferrous sulfate (FS) or iron sucrose (IS) to achieve a minimum target Hb of 11g/dl. On enrollment, a detailed clinical history was taken. Detailed physical examination including height, weight body mass index (BMI), general physical examination, and obstetrical examination was done. Antenatal care was given as per institute protocol.

Group 1

One hundred thirty-four patients were recruited into this group. The dose of IS was calculated by Ganzoni’s formula [15]. Required iron dosage in milligrams is calculated as follows: 2.4 × (target Hb - actual Hb) × pre-pregnancy weight (kg) + 500mg for body stores. An emergency tray containing injections of adrenaline, hydrocortisone, and oxygen was made available for the management of anaphylactic reactions. The general condition of the patient, blood pressure, and pulse rates were noted before infusion, and fetal heart rate monitoring was done before and after infusion. Any major or minor adverse effects were noted. The dose was given as 200mg intravenous infusion in 100ml of normal saline (NS) (0.9%) over 30 minutes thrice a week. A maximum of 600mg/week was given (maximum permissible dose in a week). They have given a tablet of folic acid 500mg once a day throughout pregnancy and six months postpartum.

Group 2 (oral ferrous sulfate)

One hundred thirty-four women were randomized into groups and were given oral iron in the form of iron and folic acid tablets available in hospital supply containing 335mg dried ferrous sulfate with 100mg elemental iron and 500mg of folic acid, twice a day for four weeks or till she achieves target Hb, and after that, she can continue with one tablet of ferrous sulfate throughout pregnancy and six months in the postpartum period. Compliance with drug intake was ensured by bringing back the empty tablet packs and asking about the change in stool color after starting treatment. All women in both groups were given standard antenatal care. A single dose of albendazole 400mg was also given.

Follow-up of patient

All women were followed up at four weeks after drug administration and at 36 weeks of gestation to check for the rise in serum ferritin and Hb. They were contacted telephonically whenever required. The method of hemoglobin estimation and other parameters are as follows: Hb and the other RBC parameters were measured using an automated hematology analyzer. Serum ferritin was measured by immune-turbidimetry using Roche kits on the Hitachi 912 clinical analyzer (Hitachi, Ramsey, Minnesota, USA). The principle of this method is that latex-bound ferritin antibodies react with antigens in the sample to form an antigen-antibody complex. The turbidity formed was proportional to ferritin concentration and was measured at 700nm. Statistical analysis of the presentation of the categorical variables was done in the form of numbers and percentages (%). On the other hand, the quantitative data were presented as the means ± SD. The following statistical tests applied to the results were that the comparison of the variables which were quantitative in nature was analyzed using the independent test. Paired t-test was used for comparison across follow-ups. The comparison of the variables which were qualitative in nature was analyzed using the Chi-Square test. If any cell had an expected value of less than 5, then Fisher’s exact test was used. The data entry was done in the Microsoft Excel spreadsheet (2019 edition, Microsoft, Redmond, Washington, USA) and the final analysis was done with the use of Statistical Package for Social Sciences (SPSS) software, IBM manufacturer, Chicago, USA, version 25.0.

## Results

The study was conducted as a parallel-group, open-label RCT in the Department of Obstetrics and Gynaecology of Shri Lal Bahadur Shastri Government Medical College & Hospital, Mandi, Himachal Pradesh. Two hundred sixty-eight pregnant women between 18 and 45 years of age with moderate IDA (Hb 7-9g/dl, microcytic-hypochromic, and serum ferritin <30ng/ml) were included in the study. Patients were randomly divided into two groups: group 1 with 134 patients to receive intravenous iron sucrose and group 2 with 134 patients to receive oral ferrous sulfate tablets.

Group 1 (intravenous iron sucrose group)

Out of 134 patients recruited in this group, 10 patients left the study after randomization. Four patients refused participation just at the start of the first dose. One hundred twenty patients participated, but eight patients had hypersensitivity reactions after the first dose and were excluded from the study. One hundred twelve patients were followed and analyzed in this group.

Group 2 (oral ferrous sulfate group)

**Figure 1:**
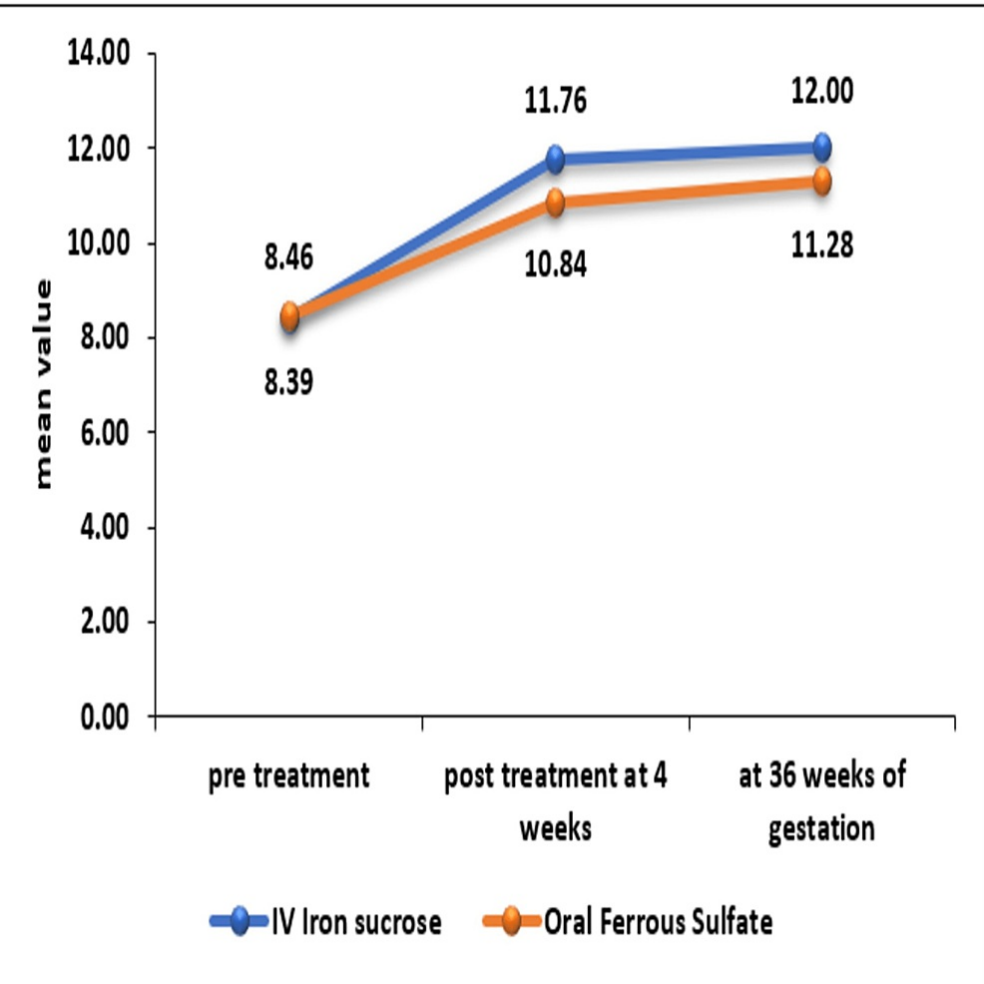
Comparison of hemoglobin (gram per deciliter) between intravenous iron sucrose and oral ferrous sulfate group

A total of 134 patients were recruited in this group, but 12 patients left the study after randomization. So a total of 122 patients were followed and analyzed in this group. The mean ± SD of age (years) of women in IV iron sucrose was 26.61 ± 4.79 and in oral ferrous sulfate was 26.39 ± 5.09 with no significant difference between them with a p-value of 0.736. The maximum number of patients falls between 21 and 29 years of age, and extremes of ages have very few patients. A majority of pregnant women recruited were vegetarian (65% and 54.92%) in the IV iron sucrose and oral ferrous sulfate group, respectively, with no statistically significant difference between the two groups. A majority of pregnant women in both groups were educated up to the 12th standard, and the difference was not statistically significant. The mean ± SD of hemoglobin (g/dL) at pretreatment in IV iron sucrose was 8.39 ± 0.5 and in oral ferrous sulfate was 8.46 ± 0.37 with no significant difference between them. A significant difference was seen in hemoglobin (g/dL) levels in post-treatment values at four weeks of therapy and 36 weeks of gestation between the IV iron sucrose and oral ferrous sulfate group with a p-value of <0.05. Post-treatment mean ± SD hemoglobin (g/dL) at four weeks of therapy and 36 weeks of gestation in the IV iron sucrose group was 11.76 ± 1.29 and 12 ± 1.1, respectively, which was significantly higher as compared to the oral ferrous sulfate group with mean ± SD of 10.84 ± 0.62g/dl at four weeks with a p-value of <0.0001 and 11.28 ± 0.59g/dl at 36 weeks of gestation with a p-value of <0.0001. A significant increase was seen in hemoglobin (g/dL) post-treatment at four weeks and 36 weeks of gestation in both the groups as compared to pretreatment with a p-value of 0.0001 as mentioned in Figure 1.

**Figure 2:**
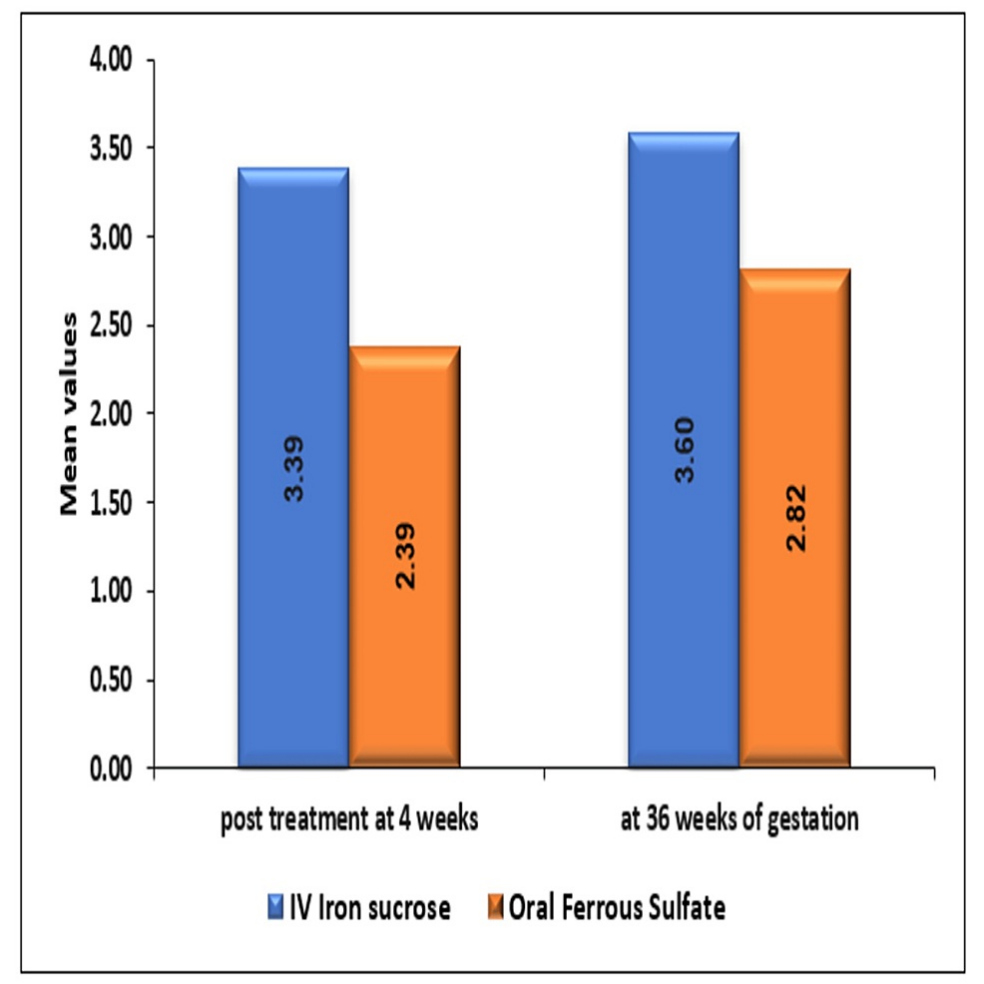
Comparison of increase in hemoglobin (gram per deciliter) between intravenous iron sucrose and oral ferrous sulfate group

The mean ± SD of increase in hemoglobin (g/dL) post-treatment at four weeks and 36 weeks of gestation in the IV iron sucrose group was 3.39 ± 1.24 and 3.6 ± 1.26, respectively, which was significantly higher as compared to the oral ferrous sulfate group where a mean increase in Hb (g/dl) was 2.39 ± 0.69 with a p-value of <0.0001 at four weeks post-treatment and 2.82 ± 0.62 with p-value <0.0001 at 36 weeks of gestation as mentioned in Figure 2.

**Figure 3:**
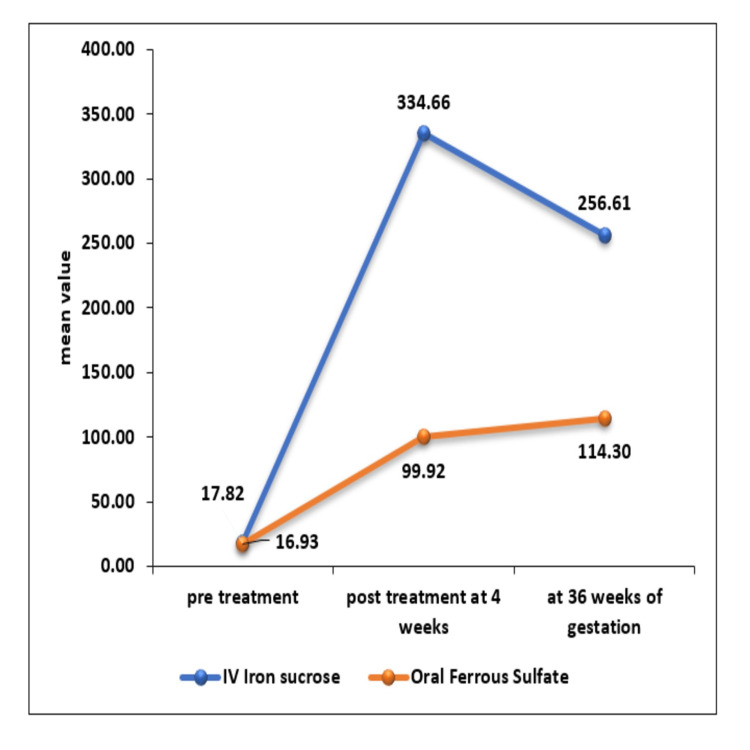
Comparison of serum ferritin between intravenous iron sucrose and oral ferrous sulfate group

The mean ± SD of serum ferritin level (ng/mL) at pretreatment in IV iron sucrose was 17.82 ± 7.41 and in oral ferrous sulfate was 16.93 ± 5.43 with no significant difference between them. A significant difference was seen in serum ferritin (ng/mL) post-treatment at four weeks and 36 weeks of gestation between the IV iron sucrose and oral ferrous sulfate group with a p-value of <0.05. The mean ± SD of serum ferritin (ng/mL) post-treatment at four weeks and 36 weeks of gestation in the IV iron sucrose group was 334.66 ± 146.57 and 256.61 ± 122.35, respectively, which was significantly higher as compared to the oral ferrous sulfate group where serum ferritin was 99.92 ± 19.34 with a p-value of <0.0001 at four weeks post-treatment and 114.3 ± 71.08 with a p-value of <0.0001 at 36 weeks of gestation. A significant increase was seen in serum ferritin (ng/mL) post-treatment at four weeks and 36 weeks of gestation in both the groups as compared to pretreatment with a p-value of <0.0001 as shown in Figure 3.

**Figure 4:**
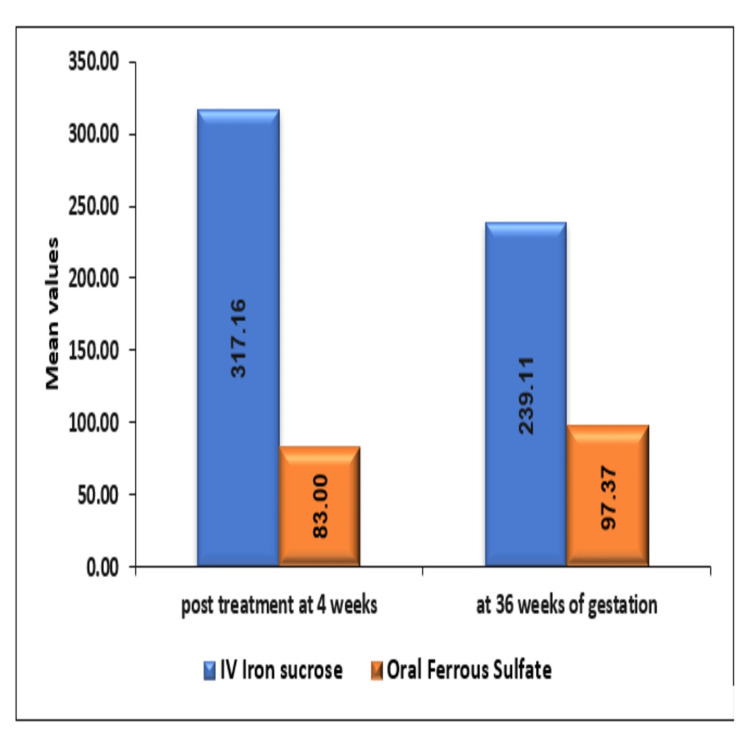
Comparison of increase in serum ferritin between intravenous iron sucrose and oral ferrous sulfate group

The mean ± SD of increase in serum ferritin (ng/mL) post-treatment at four weeks and 36 weeks of gestation in IV iron sucrose was 317.16 ± 146.19 and 239.11 ± 121.77ng/ml, respectively, which was significantly higher as compared to oral ferrous sulfate group where the increase in serum ferritin was 83 ± 18.64 with a p-value of <0.0001 and 97.37 ± 70.61 with a p-value of <0.0001, respectively, at the abovementioned period as seen in Figure 4.

The proportion of patients without complications was significantly higher in the IV iron sucrose group as compared to an oral ferrous sulfate group; 93.33% of patients had no complication in the IV iron sucrose group compared to 58.20% in the oral ferrous sulfate group. The proportion of patients with complications like nausea, gastritis, constipation, and headache was significantly lower in the IV iron sucrose group as compared to the oral ferrous sulfate group. Nausea was seen in 0% of patients in the IV iron sulfate group compared to 13.11% in the oral ferrous sulfate group; gastritis occurred in 0% of cases in IV iron sucrose as compared to 6.56% in the oral ferrous sulfate group; constipation was seen in 0% of cases of IV iron sucrose group as compared to 14.75% cases of oral ferrous sulfate group; the headache was seen in 0% of cases versus 7.38% of cases in IV iron sucrose and oral ferrous sulfate group, respectively; and 6.67% patients had mild hypersensitivity reaction in IV iron sucrose group as compared to 0% in oral ferrous sulfate group with a p-value of <0.0001.

The mean ± SD of gestational age (weeks) in IV iron sucrose was 38.54 ± 1.6 which was significantly higher as compared to oral ferrous sulfate where the mean ± SD of gestational age was 38.04 ± 1.72 with a p-value of 0.021. The mean ± SD of birth weight (grams) in the IV iron sucrose group was 2731.43 ± 303.19 and in the oral ferrous sulfate group was 2674.84 ± 293.64. There was no significant difference seen in the birth weight of neonates between the two groups with a p-value of 0.147. Patients who underwent normal delivery were 66.96% in the IV iron sucrose group as compared to 72.13% in the oral ferrous sulfate group, cesarean delivery was seen in 25.89% in the IV iron sucrose group versus 20.49% in the oral ferrous sulfate group, and instrumental delivery in 7.14% in IV iron sucrose group as compared to 7.38% in oral ferrous sulfate group with a p-value of 0.617. The number of patients who achieved target Hb was significantly higher in the IV iron sucrose group compared to the oral ferrous sulfate group with 95.54% of patients in the IV iron sucrose group compared to 72.95% in the oral ferrous sulfate group with a p-value of <0.0001.

## Discussion

Anemia remains a major health challenge during the antenatal period. IDA is the major contributor to anemia in pregnancy, so iron supplementation has been the focus of treatment of this condition for ages. The main aim of correction of anemia is to achieve an acceptable level of Hb in a maximum number of patients at the time of delivery to avoid various complications and to reduce the risk associated with blood transfusions. The present study was hence undertaken to compare intravenous iron sucrose with oral ferrous sulfate. In the present study, the mean Hb after four weeks of therapy was 11.76 ± 1.29g/dl and 10.84 ± 0.67g/dl in the IV iron sucrose and oral ferrous sulfate group, respectively. The Hb at four weeks post-treatment was significantly higher in the IV group compared to the oral group proving that IV iron sucrose corrects anemia more rapidly than an oral drug in our study. Kochhar et al. conducted RCT comparing IV and oral iron therapy [16]. At 30 days of treatment, the Hb was 12.8 ± 1.1g/dl and 10.7 ± 0.7g/dl, respectively, in two groups with a p-value of 0.002. This was comparable to the present study. In the present study, the mean Hb (g/dl) at 36 weeks of gestation was 12 ± 1.1g/dl and 11.28 ± 0.59g/dl in IV IS and oral ferrous sulfate groups, respectively. The difference between the two groups was statistically significant. So intravenous (IV) iron sucrose appears to be the better option at advanced gestation to correct anemia. As per the study conducted by Kochhar et al., Hb levels just before deliveries were 13.4 ± 0.9g/dl and 11.2 ± 0.9g/dl in intravenous IS and oral iron therapy groups, respectively [16]. Hb levels in the IV group were significantly more compared to the oral group. This was in agreement with our study.

In the present study, the rise in hemoglobin was 3.48g/dl and was significantly high in the IV iron sucrose group compared to the oral ferrous sulfate group which was 2.39g/dl after four weeks of treatment. The rise was 3.6g/dl and 2.82g/dl in the IV iron sucrose and oral ferrous sulfate group, respectively, at 36 weeks of gestation, and the difference was significant. In this study, the IV route of iron is found to be superior to the oral route. Al et al. [17] in the year 2005 conducted RCT to compare intravenous iron sucrose and oral iron polymaltose complex (300mg elemental iron per day). The mean rise in Hb from baseline in the IV group was 1.2g/dl compared to 0.6g/dl at the end of four weeks [17]. The mean rise was 2.1g/dl in the IV group compared to 1.5g/dl in the oral group at delivery. This study also concluded that the rise in Hb was significantly higher in the IV group compared to the oral group, especially at the 14th and 28th days. Intravenous iron sucrose corrected moderate IDA of pregnancy more effectively as compared to oral iron with no major side effects [17].

In the present study, the rise in ferritin was seen in both groups, but the rise was significantly higher in the IV iron sucrose group compared to the oral ferrous sulfate group after four weeks post-treatment and at 36 weeks of gestation. In the IV iron sucrose group, the mean ferritin was 334.6 ± 146.57ng/ml at four weeks after treatment, and in the oral ferrous sulfate group, the mean ferritin was 99.92 ± 19.34ng/ml [17]. At 36 weeks of gestation, the mean ferritin was 256.61 ± 122.35ng/ml and 114.3 ± 71.08ng/ml in the IV iron sucrose and oral ferrous sulfate group, respectively. The present study concluded that replenishment of stores was more prompt with intravenous iron sucrose compared to oral ferrous sulfate [17]. The same study compared intravenous iron sucrose and oral polymaltose complex (300mg elemental iron per day) in women with iron deficiency anemia [17]. They also found the serum ferritin was significantly higher in the intravenous group both at four weeks (28 ± 26µg/L versus 11 ±11µg/L in intravenous compared to oral, respectively) and at delivery (23.7 ± 13.8µg/L versus 18.1µg/L in IV and oral, respectively) [17].

In the present study, the mean rise in serum ferritin after four weeks was 317.16 ± 146.19ng/ml in IV iron sucrose and 83 ± 18.64ng/ml in the oral ferrous sulfate group, respectively. The mean rise before delivery was 239.11 ± 121.77ng/ml and 97.37 ± 70.61ng/ml, respectively, in the IV iron sucrose and oral ferrous sulfate group. The rise was seen in both groups, but the difference in the rise was significantly higher in the IV iron sucrose group compared to the oral ferrous sulfate group. Stores were replenished with IV iron sucrose more efficiently. The advantage of IV iron sucrose is the certainty of its administration to correct anemia and build stores.

In a similar study conducted by Kochhar et al. [16] in 2013, the rise in serum ferritin was 85.9 ± 8.8ng/ml and 61.1 ± 7.8ng/ml in the IV iron sucrose and oral ferrous sulfate group, respectively, at 30 days of treatment. Just before delivery, the rise in serum ferritin was 109.9 ± 11.7ng/ml and 78.1 ± 8.3ng/ml in the two groups, respectively. The result of this study indicates that iron sucrose replenishes stores better than its oral counterpart [16]. In the present study, eight patients had mild hypersensitivity reactions in the form of itching and vomiting in the IV iron sucrose group compared to 51 patients in the oral ferrous group. Therefore, IV iron sucrose appears to be a safe alternative to oral with no major side effects associated with it [16]. Elstrott et al. in their RCT found that one patient in the IV group experienced an adverse drug reaction in the form of an unpleasant taste compared to one patient who left the study in the oral group due to diarrhea as the side effect [18]. A study conducted by Shafi et al. reported an unpleasant taste as the only side effect in women receiving intravenous iron [19]. In the present study, the mean gestational age of delivery was 38.54 ± 1.6 weeks in the IV iron sucrose group compared to 38.04 ± 1.72 weeks in the oral ferrous sulfate group, and the difference was statistically significant. Although the difference in groups was statistically significant clinically, the two groups were comparable as the gestational age of delivery was around 38 weeks. This difference probably appeared due to less standard deviation and the large sample size in the present study. A study in 2002 conducted a similar RCT and found that the mean gestational age of delivery in the IV group was 37 ± 2 weeks and in the oral group was 37 ± 1 with no statistically significant difference in the two groups [18].

The mean birth weight in the present study was 2731.43 ± 303.19g in the IV iron sucrose group and 2674.84 ± 293.64g in the oral ferrous sulfate group. The incidence of low birth weight was 21.43% in the IV group and 22.13% in the oral group, and the groups were comparable. There was no difference in the birth weights of the two groups making these drugs comparable [18]. The study found that the mean birth weight was 3,595g in the IV group and 3,220g in the oral group [18]. Kochhar et al. in 2013 also found the mean birth weight in the IV group was 2870 ± 680g and 2695 ± 765g in the oral group [16].

In the present study, a majority of patients delivered by vaginal route in both groups with 66.97% in the IV group compared to 72.13% in the oral group. The rate of cesarean delivery was 25.89% in the IV iron sucrose group compared to 20.49% in the oral ferrous sulfate group. The higher rate of cesarean section in the present study can be explained by the fact that it is a tertiary care center catering to almost five districts of Himachal Pradesh. The rate of instrumental delivery was 7.14% and 7.38% in the IV iron sucrose and oral ferrous sulfate group, respectively. The mode of delivery was comparable in both groups. Kumar et al. in 2005 conducted an RCT comparing oral 100mg elemental iron daily therapy with intramuscular iron sorbitol [20]. The mode of delivery in the two groups was comparable with the majority of patients delivered by normal vaginal route (92.07% in IV and 92% in the oral group, respectively). The rate of cesarean delivery was 6.6% versus 4% in the IV and oral groups, respectively. The rate of instrumental delivery was 1.33% and 4% in the IV and oral groups, respectively, in their study. In the present study, the target Hb was 11g/dl which was reached by 107 (95.54%) patients in the IV iron sucrose group and 89 (72.95%) in the oral group at the end of treatment. A significantly higher number of patients in the IV iron sucrose group achieved target Hb compared to the oral ferrous sulfate group [20].

## Conclusions

The intravenous iron sucrose is superior in terms of tolerability and correction of iron deficiency anemia during pregnancy. It also yields a quicker rise in Hb and serum ferritin with no major side effects. In the difficult terrain of Himachal Pradesh, this makes IV iron sucrose a better option for anemic pregnant women who do not have easy access to health facilities and lack education for it resulting in a large number of them reaching hospitals with moderate to severe anemia at a later gestation. It can be given in the outpatient setting and reduces the need for blood transfusion. Therefore, intravenous iron sucrose is a safe and effective option during pregnancy.
